# Physiology of the Kidney

**Published:** 1901-07

**Authors:** Byron H. Daggett

**Affiliations:** Buffalo, N.Y.


### PHYSIOLOGY OF THE KIDNEY.

A LECTURE DELIVERED TO THE CLASS OF NURSES AT BUFFALO HOSPITAL OF THE SISTERS OF CHARITY, APRIL, 1901.

By BYRON H. DAGGETT, M. D., Buffalo, N. Y.

PHYSIOLOGY treats of function; anatomy treats of structure. The dead body, recently dead, presents the anatomy intact, its physiology, its function, its vital phenomena, its life is gone forever. Chemical forces assume sway and literally render dust unto dust.

The kidneys are the great eliminative organs of the body, constantly rejecting the poisonous waste of metabolic change. The kidneys separate from the blood excrementitious matters, which are of no further use in the economy and are simply to be discharged from the system. The kidneys, the skin and the liver are the excretory organs of the body; the liver, however, is both a secretory and an excretory organ for its product is, in part, eliminated as a waste, and in part, appropriated for further use in the system.

A secretion is formed from a membrane or gland, for use in the economy, such as serous and synovial fluids, humors of the eye and ear and spinal cord, mucus, sebaceous matter, cerumen, milk, saliva, tears, digestive juices, and the like.

An excretion is a substance separated from the blood by glandular action for elimination as a useless product.

Urine is the type of excrementitious fluids, and is retained in the bladder, as a matter of convenience. The relations of urinary excretions with nutrition and disassimilation are very interesting and important. It is well, perhaps, to preface the consideration of the special study of the physiology of the kidneys with a brief outline of the general subject. Reviewing the subject sets ajar the door of memory, through which I get a whift of my student days, and I will tell you some of the things that were told me then:

Physiology is the study of the vital phenomena of organised matter or living beings. Organised matter is arranged as it is by vital forces. Organic matter is the product of organised forces; all matter belongs to the inorganic or organic kingdoms.

The organic and inorganic bodies differ in origin, in mode of increase, in duration and in form.: (1) The inorganic is arranged by chemical forces—as a mineral; the organic is arranged by vital forces—a leaf. (2) The inorganic increases by accretion—a crystal; the organic by intussusception—a man. (3) The inorganic never had life—a stone; the organic is such by virtue of life—a tree. (4) The inorganic is never nourished—a rock; the organic grows by nourishment—a leaf. (5) The inorganic has no offspring—a pebble; the organic reproduces its kind—a fish, a man.

The organic kingdom is divided into the vegetable and animal subdivisions, and they differ as follows: (1) The vegetable appropriates inorganic matter; the animal assimilates only organic matter. (2) The vegetable absorbs matter as it finds it; the animal prepares it for absorption. 3 The vegetable assimilates and grows; the animal assimilates, grows and repairs waste. (4) The vegetable lives and grows; the animal lives, grows and perceives. (5) The vegetable grows; the animal expends its energies in growth, action, excretion and thought. The stone grows, the plant grows and lives, the animal grows, lives and perceives.

Life is a force in organised matter having power to manifest functional activity under favorable circumstances. Life then is functional activity.

The vegetable functions are common to vegetable and animal life. The animal has in addition the functions of relation, so-called perception, and the various manifestations of the nervous and muscular systems.

There are three groups of animal functions: (1) Those which relate to the growth and preservation of the individual—as prehension, mastication, insalivation, deglutition, digestion, absorption, sanguinefication, circulation, assimilation or nutrition, and excretion, and the type of an excretory organ is the kidney; (2) Those relating to the preservation of the species; (3) those of external relations, as the nervous and muscular systems.

The anatomist says that man is a bundle of tissues; the physiologist says he is a bundle of functions; the chemist says he is a compound of C, H, O, N, P, Chlo. Fl, H, Na, Ca. Mag, Fe, Mn, and Si, and the like. What is man?

The physiologist says that man is made up of four groups of proximate principles. A proximate principle or element is any substance, whether simple or compound, which exists in the body as such as can be removed without destruction—primary compounds which supply matter for others more complex.

A proximate principle: (1) is of organic origin, existing in the same form in the organic and inorganic kingdoms, as O, N, $H_2O$; (2) are non-nitrogenous elements of organic origin as starch, sugar, oil and compounds of C, H, O; (3) the tissue building or nitrogenised group composed of C, H, O, N as fibrin, albumin, stearin, cartilagin, pepsin, etc. ; (4) or excrementitious group, formed by retrograde metamorphosis (disassimilation) cholesterin, urea, creatin, creatinin (C, H, O, N) $Co_2$. The simplest form of life is a single cell. These are unilocular, animals and plants.

Man is an aggregation of cells. All the tissues of the body assume their various forms through the agency of cells. The cell is the ultimate organic principle of organised matter.

Our organs are aggregations of cells and our bodies are aggregations of organs. Upon the well-being of the cell depends the well-being of the organ, and upon the well-being of the organ depends the well-being of the body. Out of this comes cellular pathology and organic therapy. The tissues of the body are built up from cells—a cell is a minute vesicle and has five typical elements: (1) a cell wall—a thin, transparent homogeneous membrane; (2) fluid contents; (3) minute granules in the fluid; (4) a nucleus—an aggregation of granules; (5) a nucleolus, a change in the nucleus to form a new cell.

Cells originate in three ways: (1) by free cell development—in a fluid—as blood corpuscles; (2) by an equal division through the middle; (3) division of its contents.

Cells assume various forms: (1) simply enlarge, as in glands; (2) elongate and make tubes—capillaries, etc.; (3) elongate and make fibers; (4) send out processes in various directions.

Cells exist: (1) Free in the fluids of the body, as blood corpuscles; (2) attached on free surfaces, as on mucus and serous surfaces, called epithelium. They are endowed with the power of forming new material and developing new substances.

This takes us to development and the study of the physiology of the individual organs.

(*Continued in August number.*)

723 Ellicott Square.

